# Supplementary material for: Mechanisms governing the pioneering and redistribution capabilities of the non-classical pioneer PU.1
Source: Nat Commun. 2020 Jan 21;11:402. doi: 10.1038/s41467-019-13960-2 (PMC6972792; doi:10.1038/s41467-019-13960-2)
Supplement: Supplementary file 7 — Source data [file 41467_2019_13960_MOESM7_ESM.zip › Source_Data/Figure6/Figure6B_MotifScanOutput/FLI1_mutPU.1/homerResults/motif14.similar.html]

motif14

## Information for motif14

T
A
C
G
C
G
A
T
C
T
A
G
A
C
T
G
A
T
C
G
G
T
A
C
C
T
A
G
A
T
C
G
C
T
G
A
C
T
A
G
T
G
A
C
A
G
T
C
C
T
G
A
  
Reverse Opposite:  

G
A
C
T
T
C
A
G
A
C
T
G
G
A
T
C
G
A
C
T
T
A
G
C
G
A
T
C
C
A
T
G
A
T
G
C
A
G
T
C
G
A
T
C
G
C
T
A
A
T
G
C
  

|  |  |
| --- | --- |
| p-value: | 1e-90 |
| log p-value: | -2.077e+02 |
| Information Content per bp: | 1.635 |
| Number of Target Sequences with motif | 2413.0 |
| Percentage of Target Sequences with motif | 23.52% |
| Number of Background Sequences with motif | 5663.5 |
| Percentage of Background Sequences with motif | 14.86% |
| Average Position of motif in Targets | 101.2 +/- 56.1bp |
| Average Position of motif in Background | 98.4 +/- 61.4bp |
| Strand Bias (log2 ratio + to - strand density) | -0.1 |
| Multiplicity (# of sites on avg that occur together) | 1.21 |
| Motif File: | file (matrix) reverse opposite |

### Similar de novo motifs found

|  |  |  |  |  |  |  |  |
| --- | --- | --- | --- | --- | --- | --- | --- |
| Rank | Match Score | Redundant Motif | P-value | log P-value | % of Targets | % of Background | Motif file |
| 1 | 0.949 | C T G A C T A G A C T G A C T G G T A C C A T G C A T G C T A G T C A G G A T C G A C T C T G A | 1e-88 | -203.466433 | 14.01% | 7.38% | motif file (matrix) |
| 2 | 0.934 | C T A G A C T G A C T G G T C A A C T G A C T G C T A G C A T G A G T C G A T C C G T A | 1e-71 | -164.008408 | 13.47% | 7.55% | motif file (matrix) |
| 3 | 0.899 | C A T G A T G C G A T C T G A C A G T C C A G T A G T C A G T C A G T C | 1e-64 | -148.495477 | 13.43% | 7.77% | motif file (matrix) |
| 4 | 0.924 | C A G T T A C G C A G T A C T G A T C G T A C G G T A C C T A G T C A G C T G A T A C G T A G C T A G C G C T A | 1e-45 | -104.338490 | 5.73% | 2.70% | motif file (matrix) |
| 5 | 0.833 | T G C A T C G A A C T G G A T C G A T C G T A C A G T C C A T G A T G C A G T C A G T C G A T C G A C T A G C T | 1e-27 | -63.639615 | 3.72% | 1.81% | motif file (matrix) |
| 6 | 0.755 | C G T A A C T G A C T G A C T G A G T C A C T G A C T G C G T A | 1e-21 | -49.712073 | 2.48% | 1.12% | motif file (matrix) |
